# Deletion of hypoxia-inducible factor prolyl 4-hydroxylase 2 in *FoxD1*-lineage mesenchymal cells leads to congenital truncal alopecia

**DOI:** 10.1016/j.jbc.2022.101787

**Published:** 2022-03-02

**Authors:** Ann-Helen Rosendahl, Mia Monnius, Anu Laitala, Antti Railo, Ilkka Miinalainen, Ritva Heljasvaara, Joni M. Mäki, Johanna Myllyharju

**Affiliations:** 1 Oulu Center for Cell-Matrix Research, University of Oulu, Oulu, Finland; 2 Biocenter Oulu, University of Oulu, Oulu, Finland; 3 Faculty of Biochemistry and Molecular Medicine, University of Oulu, Oulu, Finland; 4 Biocenter Oulu, Electron Microscope Core Facility, University of Oulu, Oulu, Finland

**Keywords:** HIF-P4H-2, alopecia, hypoxia, hair follicle, keratin, ADAM, a disintegrin and metalloproteinase, *Bnip3*, B-cell lymphoma 2/adenovirus E1B 19 kDa interacting protein 3, cKO, conditional KO, DLL, delta-like ligand, DP, dermal papilla, E12.5, embryonic day 12.5, ES, embryonic stem, FIH, factor inhibiting HIF, *FoxD1*, Forkhead box D1, HF, hair follicle, HIF, hypoxia-inducible factor, IRS, inner root sheath, JAG, jagged ligand, KGF, keratinocyte growth factor, KRT, keratin, LOR, loricrin, NICD, Notch intracellular domain, P14, postnatal day 14, P4H, prolyl 4-hydroxylase, pO_2_, partial O_2_ pressure, SC, stem cell, SMAD, small mother against decapentaplegic, TEM, transmission electron microscopy, TGFβ, transforming growth factor β

## Abstract

Hypoxia-inducible factors (HIFs) induce numerous genes regulating oxygen homeostasis. As oxygen sensors of the cells, the HIF prolyl 4-hydroxylases (HIF-P4Hs) regulate the stability of HIFs in an oxygen-dependent manner. During hair follicle (HF) morphogenesis and cycling, the location of dermal papilla (DP) alternates between the dermis and hypodermis and results in varying oxygen levels for the DP cells. These cells are known to express hypoxia-inducible genes, but the role of the hypoxia response pathway in HF development and homeostasis has not been studied. Using conditional gene targeting and analysis of hair morphogenesis, we show here that lack of *Hif-p4h-2* in Forkhead box D1 (*FoxD1*)-lineage mesodermal cells interferes with the normal HF development in mice. *FoxD1*-lineage cells were found to be mainly mesenchymal cells located in the dermis of truncal skin, including those cells composing the DP of HFs. We found that upon *Hif-p4h-2* inactivation, HF development was disturbed during the first catagen leading to formation of epithelial-lined HF cysts filled by unorganized keratins, which eventually manifested as truncal alopecia. Furthermore, the depletion of *Hif-p4h-2* led to HIF stabilization and dysregulation of multiple genes involved in keratin formation, HF differentiation, and HIF, transforming growth factor β (TGF-β), and Notch signaling. We hypothesize that the failure of HF cycling is likely to be mechanistically caused by disruption of the interplay of the HIF, TGF-β, and Notch pathways. In summary, we show here for the first time that HIF-P4H-2 function in *FoxD1*-lineage cells is essential for the normal development and homeostasis of HFs.

Cells have an intrinsic response to low O_2_ concentrations that is controlled by the hypoxia-inducible transcription factor (HIF, αβ dimers). The HIFα subunits are negatively regulated by the HIF prolyl 4-hydroxylases 1 to 3 (HIF-P4Hs 1–3, see alternative nomenclature in Table S1) in normoxia (1, 2, 3, 4, 5), HIF-P4H-2 being the main isoform regulating HIFα availability (6). In normoxia, the HIF-P4Hs hydroxylate HIFα targeting it for proteasomal degradation, whereas in hypoxia, the O_2_-dependent HIF-P4Hs are inhibited, which leads to accumulation of HIFα, and induction of the hypoxia response pathway by upregulation of HIF target genes (7, 8). Although several substrates besides HIFs have been suggested for the HIF-P4H-2, none of them seemed to have neither high reactivity with recombinant prolyl hydroxylase (9) nor relevant link to hair follicle (HF) morphogenesis or cycling.

Physiological hypoxia is vital for morphogenesis of tissues (10, 11). In mature skin, partial O_2_ pressure (pO_2_) ranges between 0.2 and 10%, and in HFs, pO_2_ is 0.1 to 0.8% (12), however, pO2 is potentially higher in sebaceous glands and dermal papilla (DP) since they are in close proximity with the dermal capillaries providing oxygen. Furthermore, during the HF morphogenesis and cycling, the location of DP alternates between the dermis and hypodermis and may thus provide temporally varying oxygen levels for the cells (13). HIF1α protein is abundant in HFs and sebaceous glands and is present at low levels in the basal keratinocyte layer (14, 15, 16). HIF1α enhances keratinocyte and dermal fibroblast mobility and promotes cell proliferation and survival (14). HIF2α is sporadically seen in the dermal area of the skin (16). It is also localized in the HF bulb precortex area above the matrix cells and is involved in the hair production and cell differentiation in HFs (17).

In mammals, HFs have an ability to regenerate in cycles of growth (anagen), involution (catagen), and rest (telogen). This regenerative potential is plausible because of a reservoir of multipotent stem cells (SCs) located in the HF bulge area (18). Morphogenesis of mouse HFs starts at embryonic day 12.5 (E12.5) with the patterning of perfectly ordered pregerm patches in the epidermis (19). At E14.5, epidermal signaling from the epidermal thickening (*i.e.*, placode) forces mesenchymal fibroblasts to accumulate underneath the placodes and to form a dermal condensate that will later differentiate into DP cells of the mature HF that forms around 2 weeks after birth. The HF morphogenesis can be seen as an anagen-like growth phase, after which the HF proceeds to the catagen phase of its first HF cycle (20, 21, 22).

HF SCs express hypoxia-inducible genes (23), but the role of hypoxia signaling in HF development and homeostasis has not been elucidated. In this study, we inactivated the main HIF regulator *Hif-p4h-2* in Forkhead box D1 (*FoxD1*)-lineage mesenchymal cells in mice. This resulted in postnatal truncal alopecia, with a premature catagen initiation and epidermal cyst formation. We show here for the first time that HIF-P4H-2 regulation of hypoxia signaling is crucial for the development and homeostasis of the hair and HF cycling.

## Results

### Inactivation of *Hif-p4h-2* in *FoxD1*-lineage dermal cells results in truncal congenital alopecia

**Figure 1:**
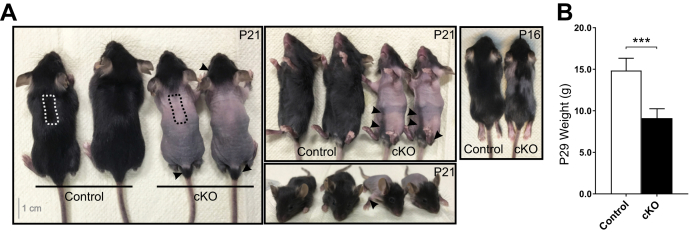
**Inactivation of *Hif-p4h-2 in FoxD1*-lineage dermal cells causes congenital truncal alopecia.***A*, the cKO mice have progressive congenital truncal alopecia but maintain normal hair on the head and neck and have also traces of hair around the ankles, tail base, and on the abdominal side of the body (*arrowheads*). Skin biopsies for further analyses were taken from the upper back skin region indicated with a *dashed line*. *B*, weight difference between control (n = 6) and cKO (n = 4) mice at P29. Data are presented as mean ± SD. ∗∗∗*p* < 0.001. cKO, conditional KO; *FoxD1*, Forkhead box D1; HIF-P4H, hypoxia-inducible factor prolyl 4-hydroxylase; P29, postnatal day 29.

A conditional *Hif-p4h-2* (*Hif-p4h-2*^*loxP/loxP*^) mouse line was generated as described in the Experimental procedures section. To examine the role of HIF-P4H-2 in the dermal *FoxD1*-lineage cells, we crossbred transgenic *FoxD1-Cre* (*FoxD1*^*Cre/+*^) mice with *Hif-p4h-2*^*loxP/loxP*^ mice to obtain conditional *Hif-p4h-2*^*loxP/loxP*^*;FoxD1*^*Cre/+*^ KO (cKO) mice (Fig. S1, *A*–*F*). Truncal hair was lost in the cKO mice, and their size was smaller (Fig. 1, *A* and *B*). Cranial hair of the cKO mice was normal, and some hair existed around the ankles, around the tail base, and sparsely on the abdominal side of the body (Fig. 1*A*). Formation of whiskers, nails, and teeth was apparently normal in the cKO mice. None of the other littermate genotypes obtained from the matings displayed the alopecia phenotype and were used as littermate controls in the following analyses. Besides alopecia, the cKO animals developed polycythemia (Fig. S1, *G*–*I*), as described previously (24).

### Inactivation of *Hif-p4h-2* in *FoxD1*-lineage cells leads to disruption of HF cycling and formation of epidermal cysts

**Figure 2:**
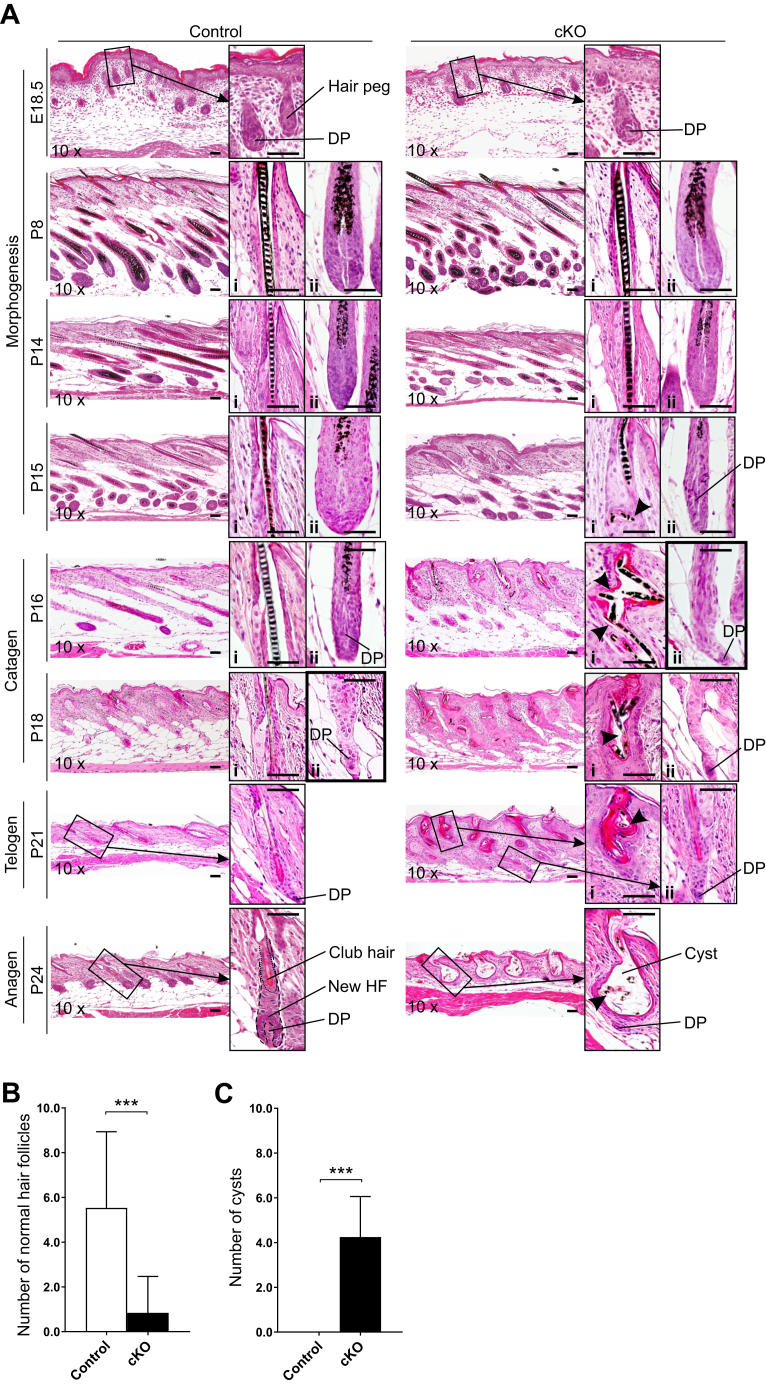
**Inactivation of *Hif-p4h-2* in *FoxD1-*lineage dermal cells disturbs skin and HF homeostasis and causes epidermal cyst formation and premature catagen induction.***A*, H&E staining of dorsal skin of control and cKO mice at indicated time points. Normal HF stages are indicated on the *left side* of the images. The first magnified inset (i) shows the infundibulum and isthmus area of the HF, whereas the second inset (ii) shows the bulb area of the HF. Breaking of the hair shaft to small pieces is indicated with *arrowheads* in the cKO mice. A late-stage catagen phase is indicated in the HF bulb insets (ii) with a thicker frame. At the initiation of a new anagen phase at P24, the control HF is showing one club hair and the beginning of a new HF, which is not occurring in the cKO mice. *B*, number of normal HFs per four visual fields/mouse in control (n = 15) and cKO (n = 18) mice (P21, P27, P29, an P33). *C*, number of cysts per four visual fields/mouse in control (n = 37) and cKO (n = 35) mice (P21, P24, P27, P29, and P33). Data are presented as mean ± SD. ∗*p* < 0.05, ∗∗∗*p* < 0.001. The scale bars represent 50 μm. cKO, conditional KO; *FoxD1*, Forkhead box D1; HF, hair follicle; HIF-P4H, hypoxia-inducible factor prolyl 4-hydroxylase; P24, postnatal day 24.

HF morphogenesis at E14.5–postnatal day 14 (P14) occurred normally in the cKO mice (Fig. 2*A*). First, differences were observed at P15 in the cKO HFs as a failure to maintain the integrity of the upper permanent part of the HF (Fig. 2*A*). Subsequently, at P16, the HF bulb of the cKO mice had progressed to a late-stage catagen, whereas the control mouse HF reached a similar stage 2 days later (Fig. 2*A*). As the HF cycle progressed, the superficial part of the HFs in the cKO mice failed to maintain its structure and rigidity. The hair formation failed, and the infundibulum and isthmus of the HF expanded to an epidermal cyst filled with keratin (KRT) and hair fragments (Figs. 2*A* and S2). At P24, the first anagen started normally in the control mice by creating a new HF that engulfs the DP cells, whereas the club hair, ensuring coating at all times, remained from the morphogenesis and rested in its own silo in the HF upper part (Fig. 2*A*) (25). Neither club hair formation nor initiation of a new anagen phase was observed in the P24 HFs of the cKO mice (Fig. 2*A*). From the beginning of telogen of the first HF cycle (P21), the number of normal HFs in the cKO mice was significantly reduced and epidermal cysts were prevalent (Figs. 2, *A*–*C* and S2). The cysts were located in the upper part of the HF (Fig. 2*A*), mainly comprising infundibulum and isthmus, which are the permanent parts of the HF, and not involved in the HF cycling.

### *FoxD1*-lineage cells are located in the truncal dermis and DP of the HF

**Figure 3:**
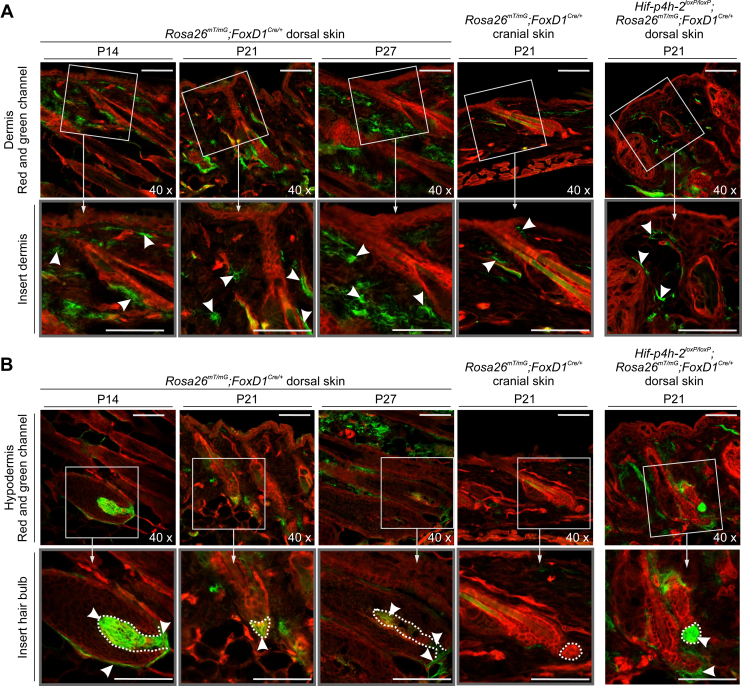
**Distribution of *FoxD1-Cre*–expressing cells in mouse skin.***A*, dorsal dermis at P14 (morphogenesis, stage 8), P21 (telogen), and P27 (anagen) and cranial skin dermis at P21. *B*, the dorsal hypodermis and hair bulbs at P14, P21, and P27 and the hair bulb in cranial hypodermis at P21. Numerous *green FoxD1*^+^ cells (indicated with arrowheads in the magnified insets) are observed in the dorsal dermis, whereas they are rare in the cranial dermis. The location of dermal papilla (DP) is indicated with a *dashed line* in *B*. The scale bars represent 50 μm. *FoxD1*, Forkhead box D1; P14, postnatal day 14.

According to the gene expression library of mouse HFs (http://hair-gel.net/, see Refs. (26, 27) for further details), expression of *FoxD1* is considerably high in dermal fibroblasts and DP cells at P5 (Fig. S3*A*) and virtually absent from most of the other cell lines of the skin. According to the same database, HIF-P4H-2 is the major and most abundant isoenzyme in mouse P5 HF cell types and in skin *in toto* (Fig. S3, *B*–*D*). Both *Hif-p4h-2* and *Hif1a* were expressed in the same cell types as *FoxD1*, and their expression was abundant also in all other cell types studied. To confirm these data and to identify the distribution of the *FoxD1*-lineage mesenchymal cells in the skin, we crossbred the *FoxD1*^*Cre/+*^ mice with double fluorescent *Cre* reporter mice, the *Rosa26*^*mT/mG*^ mice. *FoxD1*-*Cre*–mediated deletion was observed in dermal cells with a fibroblast-like morphology, as well as in the DP cells in the HFs at P14 (morphogenesis), P21 (first telogen), and P27 (early first anagen) (Fig. 3). In the cranial P21 skin sections, only a few *FoxD1*^+^ cells were observed in the dermis and no *FoxD1*^+^ DP cells were detected in the HF (Fig. 3). These observations are supported by studies showing that the cells in head and neck dermis originate from the neural crest of the ectoderm (28), whereas the cells in ventrolateral and dorsal dermis are of mesodermal origin (20, 29). We also confirmed the localization of *FoxD1*-lineage cells in the cKO mice by producing cKO mice that express simultaneously the *Rosa26*^*mT/mG*^ and *FoxD1-Cre* transgenes. The localization of *FoxD1*^*+*^ cells was not affected by *Hif-p4h-2* deletion (Fig. 3). The difference in the origin and, hence *FoxD1* expression in the cranial and truncal dermal fibroblast–like cells, thus explains the distribution of the alopecia in the cKO animals and underlies the importance of HIF-P4H-2 in hair development.

### Inactivation of *Hif-p4h-2* in *FoxD1*-lineage cells leads to activation of the hypoxia response pathway in the skin

**Figure 4:**
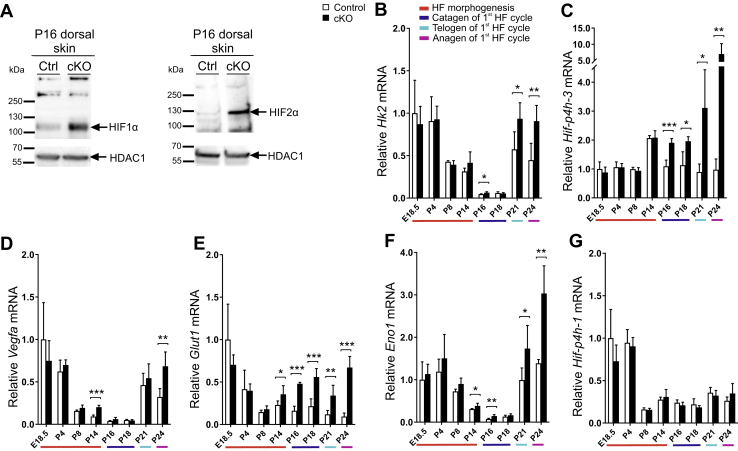
**HIF1α and HIF2α are stabilized, and expression of HIF target genes is upregulated in cKO skin.***A*, Western blot analysis of HIF1α and HIF2α in control and cKO P16 skin. Histone deacetylase 1 (HDAC1) was used as a loading control. *B*–*G*, quantitative PCR (qPCR) analysis of mRNA expression of HIF target genes *Hk2* (*B*), *Hif-p4h-3* (*C*), *Vegfa* (*D*), *Glut1* (*E*), *Eno1* (*F*), and the non-HIF target *Hif-p4h-1* (*G*) at indicated time points. The colors beneath the bar charts indicate the HF cycle stages, n = 4 to 7 per genotype. Data are presented as mean ± SD. ∗*p* < 0.05, ∗∗*p* < 0.01, and ∗∗∗*p* < 0.001. cKO, conditional KO; HIF, hypoxia-inducible factor; HIF-P4H, HIF prolyl 4-hydroxylase.

To confirm the inactivation of *Hif-p4h-2* in dermal *FoxD1*-lineage cells, we analyzed the protein expression of HIF-P4H-2, HIF1α, and HIF2α from the cKO and control mouse skin (P16). Inactivation of the *Hif-p4h-2* gene in the skin is solely present in the mesenchymal cells derived from the *FoxD1* lineage, which represent only a fraction of the total cell amount in the skin (Fig. 3). Nevertheless, a decreased amount of HIF-P4H-2 protein was observed in cKO skin *in toto* (Fig. S1, *E* and *F*), and HIF1α and HIF2α were markedly stabilized in the cKO skin (Fig. 4*A*). Furthermore, a systematic mRNA upregulation of known HIF target genes was observed in the cKO skin from P14 to P16 onward (Fig. 4, *B*–*F*), whereas no difference was observed in the mRNA level of a nontarget gene *Hif-p4h-1* (Fig. 4*G*).

### Inactivation of *Hif-p4h-2* in *FoxD1*-lineage cells leads to upregulation of genes involved in skin barrier function and hair formation

**Figure 5:**
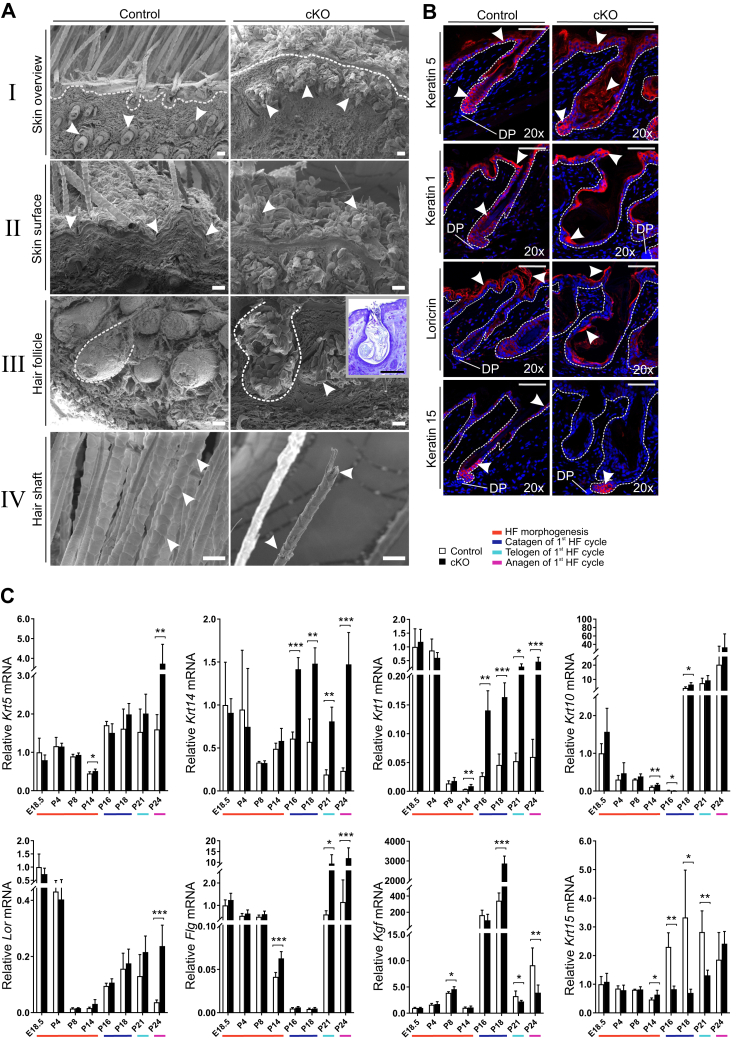
**Inactivation of *Hif-p4h-2* in *FoxD1*-lineage dermal cells increases the expression of keratins and changes their distribution in the skin.***A*, scanning electron microscopy analysis of P24 control and cKO skin. I: HFs are indicated with *arrowheads*, and the basement membrane is visualized with a *dashed line*. In cKO skin, keratin-filled cysts mainly located in the upper part of the dermis are observed. II: keratins on the skin surface are indicated with *arrowheads*. They separate from the skin in control mice as sheaths, whereas they emerge as clusters on the cKO skin. III: HFs are indicated with a *dashed line*. An *arrowhead* indicates a fragment of a hair shaft inside a cyst in cKO skin. The cKO inset shows the histological appearance of a similar cyst. IV: the hair shaft scaling is indicated with *arrowheads*. The cKO skin hair shaft scaling is irregular, and the hair shaft is fragile and breaks easily. The scale bars represent 20 μm, except in the inset figure in *A* (III) 50 μm. *B*, immunofluorescence staining of keratins 1, 5, and 15 and loricrin in control and cKO dorsal skin at P21. Examples of positive signals are indicated by *arrowheads*. Dermal papilla (DP) is shown in the figures, and the basement membrane is visualized with a *dashed line*. The scale bars represent 50 μm. *C*, quantitative PCR (qPCR) analysis of mRNA expression of keratins (*Krt5*, *Krt14*, *Krt1*, *Krt10*, and *Krt15*), loricrin (*Lor*), filaggrin (*Flg*), and keratinocyte growth factor (*Kgf*) in control and cKO mouse skin. The colors beneath the bar charts indicate the HF cycle stages, n = 4 to 7 mice per genotype. Data are presented as mean ± SD. ∗*p* < 0.05, ∗∗*p* < 0.01, and ∗∗∗*p* < 0.001. cKO, conditional KO; *FoxD1*, Forkhead box D1; HF, hair follicle; HIF-P4H, hypoxia-inducible factor prolyl 4-hydroxylase; P24, postnatal day 24.

We next analyzed the cKO skin structure and epidermal cyst formation in more detail by scanning electron microscopy, which showed that the skin had an abnormal appearance, with sparse and irregular hair formation and atypical KRT shedding from the skin surface (Fig. 5*A*). The cysts in the cKO skin contained accumulated KRT and small pieces of broken hair shafts and were typically located in the upper part of the dermis. Only a few fragile and irregular hair shafts existed in the cKO skin (Fig. 5*A*). We next studied the expression and distribution of various KRTs and KRT-related proteins in cKO skin. Proliferative progenitor cells in the epidermal basal layer and the outer root sheath are rich in dimerized KRT5 and KRT14 (30, 31, 32, 33), which provide mechanical support and cytoprotection in the basal cells (33). The basal cells are responsible for the integrity of the epidermal basal layer and are the origin of the nonproliferative upper layers of the skin (31). The spinous and granular cell layers of the epidermis express dimerized KRT1 and KRT10, which have a major role in epidermal barrier formation (30, 34). As the keratinocytes differentiate to a postmitotic stage and migrate toward the skin surface, *Krt5*/*Krt14* expression is reduced and *Krt1*/*Krt10* expression is induced (33). The cysts in the cKO skin were filled with KRT5 (Fig. 5*B*), and its staining in the skin was diffused in the epidermis and cyst edge, whereas in the controls, the staining was specific to the epidermal basal cells and to the HF (Fig. 5*B*). *Krt5* expression was significantly increased in the cKO mice at the end of morphogenesis (P14) and at the beginning of anagen of the first HF cycle (P24) but was unaffected in other time points (Fig. 5*C*). *Krt14* mRNA level was significantly upregulated in cKO mouse skin starting from P16, the higher expression level persisting during the remaining HF cycle (Fig. 5*C*). The upper epidermal layer was intensely stained with KRT1 in the cKO skin (Fig. 5*B*). KRT1 was localized on the edges of the cysts, suggesting that the cKO mice attempt to develop the inner root sheath (IRS) of the HFs (Fig. 5*B*). In cKO mice, a premature induction of *Krt1* expression was observed at P14, and it persisted throughout the cyst formation, whereas less pronounced effects on *Krt10* expression were observed at P14 to P18 (Fig. 5*C*).

Loricrin (LOR) is expressed in the IRS and *stratum corneum* and contributes to the protective barrier of the cornified envelope (30). The apical side of the cysts of the cKO mice produced LOR (Fig. 5*B*). LOR was also localized on the outermost surface of the skin in both genotypes (Fig. 5*B*). No differences were observed in *Lor* mRNA expression at E18.5–P21, but at the induction of anagen of the first HF cycle (P24), *Lor* expression was maintained at a higher level in the cKO mice (Fig. 5*C*). Filaggrin is necessary not only for the formation and continuance of the cornified envelope by binding to KRTs but also for the flattening of the cells in the cornified layer (35). Filaggrin mRNA expression was higher in the cKO at P14, normalized during catagen, and enhanced again during telogen (P21) and beginning of anagen (P24) of the first HF cycle, relative to control (Fig. 5*C*).

### Inactivation of *Hif-p4h-2* in *FoxD1*-lineage cells leads to abnormal expression of keratinocyte growth factor and KRT15 and disturbs cornification of the IRS

DP cells influence the HF bulge SCs to progress to the next phase of the HF cycle (31). We examined whether inactivation of *Hif-p4h-2* in the DP cells affects their communication with the bulge SCs, which could result in the interruption of the development of the HF and subsequent cyst formation. Hypoxia-inducible keratinocyte growth factor (KGF) is produced by mesenchymal fibroblasts to ensure the colony formation and proliferation of the overlying keratinocytes. Furthermore, the DP cells produce KGF during the initiation of anagen to influence the matrix keratinocytes to create a new HF (36, 37). At the end of the catagen (P18), a strong upregulation of *Kgf* expression was observed in the cKO mice relative to control, which later at P21 and P24 converted to a marked downregulation of the *Kgf* mRNA (Fig. 5*C*). This indicates that the initiation of the anagen phase by the cKO DP cells in the cyst bulge SCs is disturbed.

The bulge SC marker KRT15 is coproduced with the KRT5–KRT14 heterodimer in the basal layers of the epidermis (38). In cKO P21 HFs, KRT15^+^ cells (*i.e.*, potential SCs) were located specifically in the bulb area of the cyst, whereas in the controls, faint staining of KRT15^+^ cells was detected within the basement membrane of the epidermis and in the bulge (Fig. 5*B*). In some of the cKO hair cysts, KRT15^+^ staining was completely absent, whereas in other cysts, smaller clusters of KRT15^+^ staining were observed. The *Krt15* mRNA level was significantly higher at P14 in the cKO skin, whereas during the HF catagen and telogen (P16–P21), the level was significantly reduced relative to controls (Fig. 5*C*). These data suggest that the bulge SC population is abnormally distributed in the cKO mice, causing interference in the signaling from the DP cells to the bulge SCs.

**Figure 6:**
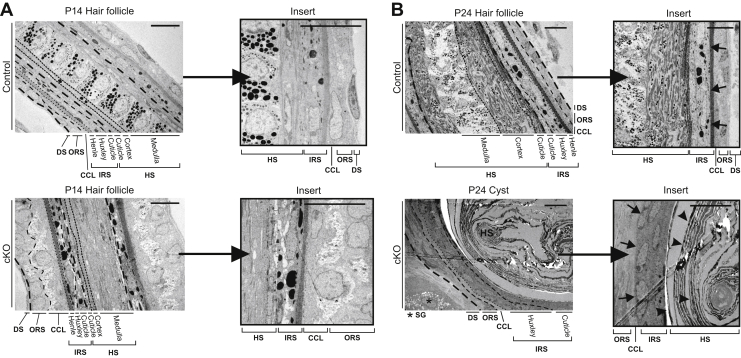
**Epidermal cysts in the cKO mouse skin are poorly differentiated and do not develop Henle’s layer of the inner root sheath.** Transmission electron microscopy analysis of control HF and cKO cysts at P14 (*A*) and P24 (*B*). Cell layers are separated with *dashed lines*. The control HF Henle’s layer is indicated in the P24 (*B*) magnified inset with *arrows*. In the P24 cKO inset, the *arrows* indicate the place where Henle’s layer should develop. *Arrowheads* show keratin peeling off the sides of the cyst as layers creating the keratin mass inside the cyst. The scale bars represent 10 μm. CCL, companion cell layer; cKO, conditional KO; Co, cortex; Cu, cuticle; DS, dermal sheath; He, Henle’s layer; HF, hair follicle; HS, hair shaft; Hu, Huxley’s layer; IRS, inner root sheath; Me, medulla; ORS, outer root sheath; P14, postnatal day 14; SG, sebaceous gland.

Transmission electron microscopy (TEM) revealed that formation of both the hair shaft and the HF was disturbed in the cKO mice. In a cross-section of control P14 HF and hair shaft, all cell layers were visible (Fig. 6*A*). In the cKO P14 HF and hair shaft, the same cell layers were present, but they had morphological discrepancies. The Henle’s layer in the cKO IRS was not as uniform in thickness as in the control, and the cKO outer root sheath layers contained unidentified loose material in the cytoplasm. The cells also seemed to have lost their polarity and normal morphology. The P24 cKO cysts were composed of an unstructured mass of hair shaft medulla, cortex, and cuticle layers that were not adhering to each other normally but were peeling off from the HF structure (Fig. 6*B*). Moreover, the IRS of the cKO mice completely lacked the Henle’s layer (Fig. 6*B*), which is an important structure as it is the first of the IRS layers to cornify as the cells travel up from the bulb of the HF and mature (39). It molds and protects the hair shaft and forms a cornified sheet around the HF to keep its structure intact (40).

### Inactivation of *Hif-p4h-2* in *FoxD1*-lineage cells does not affect apoptosis and proliferation of HF keratinocytes

Proliferation and apoptosis of HF cells are strictly regulated during HF cycling (41, 42). No difference between the genotypes was observed in the proliferation of P21 HF keratinocytes (Fig. S4, *A* and *B*). Since the diameter of HFs is consistent with an increase or a decrease in HF proliferation (17), we also measured the diameter of the HFs, but no differences were observed between the genotypes (data not shown). Analysis of apoptotic keratinocytes by TUNEL assay in P21 HFs showed no differences between the genotypes (Fig. S4, *C* and *D*). Despite this, we analyzed the expression level of the B-cell lymphoma 2/adenovirus E1B 19 kDa interacting protein 3 (*Bnip3*), an HIF target gene (43, 44) that has been linked to increased autophagocytosis besides apoptotic activity (45). We found a significantly increased *Bnip3* mRNA level in the cKO skin at P8, P14, and P24 (Fig. S4*E*).

### Inactivation of *Hif-p4h-2* in *FoxD1*-lineage cells leads to upregulation of transforming growth factor β signaling in HFs

**Figure 7:**
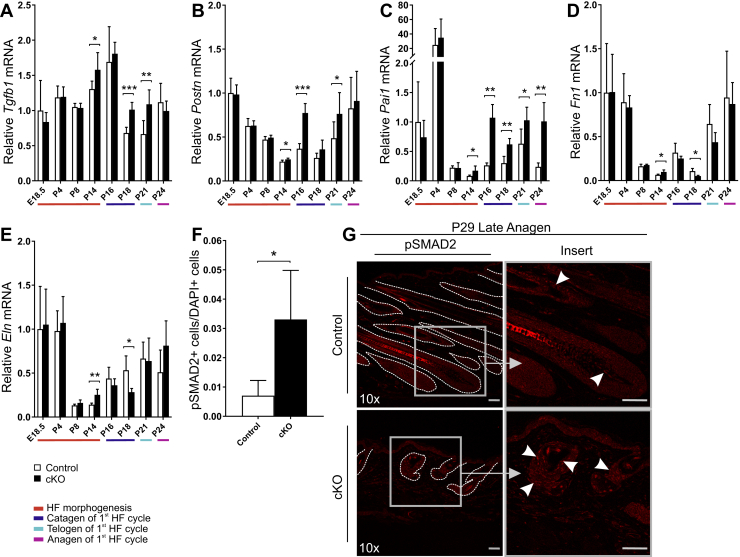
**TGFβ signaling is upregulated in the cKO skin.** Quantitative PCR (qPCR) analysis of mRNA expression of *Tgfb1* (*A*) and the TGFβ target genes *Postn* (*B*), *Pai1* (*C*), *Fn1* (*D*), and *Eln* (*E*) at indicated time points. The colors beneath the bar charts indicate the HF cycle stages, n = 4 to 7 per genotype. *F*, morphometric analysis of pSMAD2^+^ keratinocytes in the HF. Control (n = 5) and cKO (n = 6). Data are presented as mean ± SD. ∗*p* < 0.05, ∗∗*p* < 0.01, and ∗∗∗*p* < 0.001. *G*, representative images of immunostaining of pSMAD2 in the skin. *Arrowheads* indicate pSMAD2 staining in the magnified insets. HF structures in the control and cysts in the cKO mouse skin are indicated with a *dashed line*. The scale bars represent 50 μm. cKO, conditional KO; HF, hair follicle; TGFβ, transforming growth factor β.

Transforming growth factor β (TGFβ) has an important role in HF development and cycling (46). TGFβ1 induces anagen by activating apoptosis and reducing keratinocyte proliferation, whereas TGFβ2 has been linked to the induction of the HF growth during morphogenesis, and both of them have been implicated in the anagen–catagen switch (47, 48). We observed differential mRNA expression patterns of the TGFβ isoforms as well as their target genes in the cKO and control mouse skin from P14 onward (Figs. 7, *A*–*E* and S5, *A*–*D*). The mRNA expression of TGFβ1 was typically upregulated (Fig. 7*A*) in most of the time points, whereas TGFβ2 was downregulated and TGFβ3 was mostly unchanged (Fig. S5, *A* and *B*). Since, especially, periostin (*Postn*) and plasminogen activator inhibitor 1 (*Pai1*), target genes of TGFβ signaling, showed significant and parallel changes in their expression patterns (Figs. 7, *B*–*E* and S5, *C* and *D*), we analyzed small mother against decapentaplegic (SMAD) 2 phosphorylation within the HF. Phosphorylation of SMAD2 was significantly higher in the cKO HF cysts at late anagen (P29) when compared with the control HFs (Fig. 7, *F* and *G*), indicating upregulation of canonical TGFβ signaling in the cKO HF cysts.

### Inactivation of *Hif-p4h-2* in *FoxD1*-lineage cells disturbs Notch signaling in the skin

**Figure 8:**
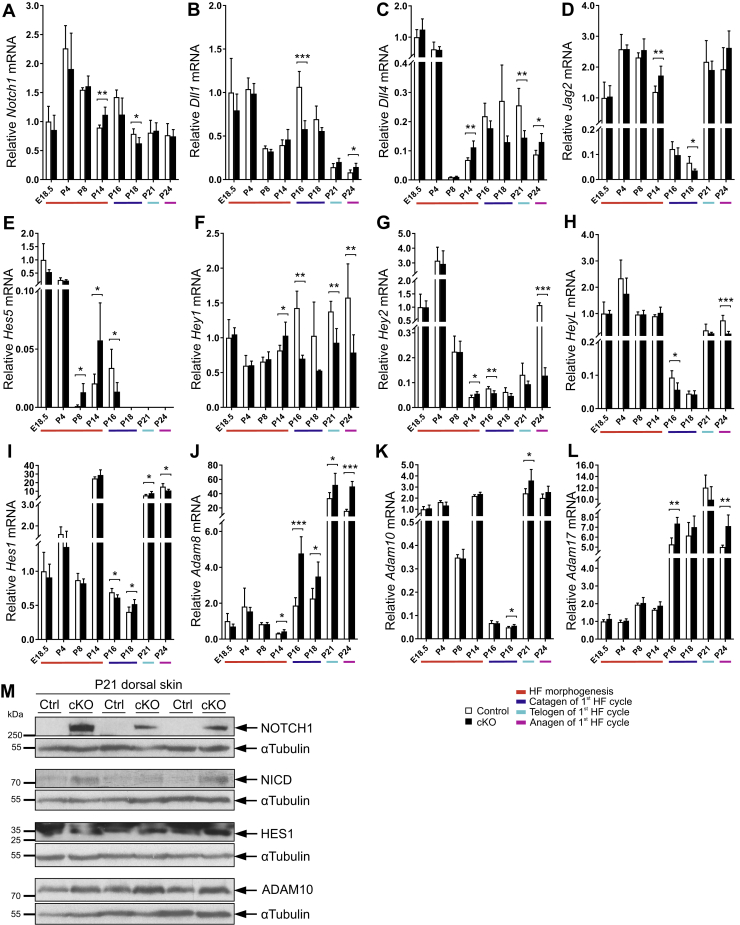
**Notch signaling is disturbed in the cKO skin.** Quantitative PCR (qPCR) analysis of mRNA expression of *Notch1* (*A*); NOTCH ligands *Dll1*, *Dll4*, and *Jag2* (*B–D*); NOTCH target genes *Hes5*, *Hey1*, *Hey2*, *HeyL*, *and Hes1* (*E–I*); the ADAM metalloproteinases *Adam8*, *Adam10*, and *Adam17* (*J–L*) at indicated time points. The colors beneath the bar charts indicate the HF cycle stages, n = 4 to 7 per genotype. *M*, Western blot analysis of NOTCH1, NICD, HES1, and ADAM10 protein in P21 dorsal skin samples. Data are presented as mean ± SD. ∗*p* < 0.05, ∗∗*p* < 0.01, and ∗∗∗*p* < 0.001. ADAM, a disintegrin and metalloproteinase; cKO, conditional KO; HF, hair follicle; P21, postnatal day 21.

Notch is an important factor for postnatal HF development and homeostasis (49). Notch is expressed abundantly both in the HF and the epidermis together with its activating ligands (50, 51). The Notch receptor ligands, such as delta-like ligands (DLL1, DLL3, and DLL4) and jagged ligands (JAG1 and JAG2), can activate Notch signaling by ligand–receptor interaction and are expressed in Notch receptor neighboring cells (50). Furthermore, a disintegrin and metalloproteinase (ADAM) metalloproteinases, that is, ADAM8, ADAM10, and ADAM17, can activate Notch signaling by extracellular cleavage, whereas γ-secretase releases the Notch intracellular domain (NICD, the activated Notch) in the cells, which can subsequently activate Notch target genes (52, 53). We therefore analyzed the mRNA levels of the Notch receptors 1 to 4 (*Notch1–4*), their ligands and other activators such as selected ADAMs, and Notch target genes from the skin samples. We found fluctuating differences in the relative expression levels of these genes between the genotypes mostly starting around P14 (Figs. 8, *A*–*L* and S5, *E*–*I*). At the protein level, the amount of NOTCH1 and its activated form NICD, as well as its target hairy and enhancer of split-1 (HES1) and potential activator ADAM10, were clearly increased in the cKO mouse skin at P21 (Fig. 8*M*). Interestingly, factor inhibiting HIF (FIH) is known to hydroxylate NICD and inhibit its function (41). FIH mRNA level was similar between the genotypes until P24, when its mRNA level was significantly higher in the cKO mouse skin (Fig. S5*J*). Interestingly, higher FIH protein amount was seen in cKO skin already at P14 (Fig. S5*K*). Taken together, the data suggest that Notch signaling homeostasis is disturbed in the cKO mice.

## Discussion

We show here for the first time that inactivation of the main oxygen sensor HIF-P4H-2 in *FoxD1*-lineage cells disrupts normal HF development and cycling. Global inactivation of HIF-P4H-2 in mouse leads to death of the embryos at E12.5 to E14.5 (54), whereas global conditional inactivation of HIF-P4H-2 by tamoxifen administration 17.5 days after coitus and at 3 weeks of age leads to polycythemia and congestive heart failure (55). Polycythemia is also observed when HIF-P4H-2 is inactivated in the *FoxD1* lineage (24 and this study), as the erythropoietin-producing kidney tubular interstitial fibroblasts are also derived from the *FoxD1* lineage. However, alopecia has not been reported in the previous studies. A similar hairless phenotype has been shown in mice lacking ADAM10, NOTCH1, recombination signal–binding protein for immunoglobulin kappa J region, and γ-secretase, activators of the Notch signaling pathway (49, 53, 56). Like our cKO mice, these mice display an undisturbed early postnatal HF formation and have a premature catagen initiation and epidermal cyst formation.

In our cKO mice, most of the mRNA level changes of the analyzed genes started around P14 in late morphogenesis and manifested as a progressive HF phenotype starting from P15, eventually leading to alopecia caused by a premature catagen initiation and epidermal cyst formation. The observed changes most probably result from altered communication between the mesenchymal and epithelial cells, since *Hif-p4h-2* is not deleted in keratinocytes, which are responsible for producing multiple types of KRTs and KRT-related proteins needed for proper hair formation. From a mechanistic point of view, besides the HIF pathway, we found differential regulation of two major signaling pathways regulating the HF cycling, the Notch, and TGFβ pathways.

Our results show that inactivation of HIF-P4H-2 changes Notch signaling in skin as shown by the differential expression of its receptors, target genes, ligands, and other activating factors such as ADAMs (Figs. 8 and S5). At protein level, at P21 (first telogen), NOTCH1 is virtually absent in control skin, whereas it is upregulated strongly in cKO skin, and upregulation of the activated Notch (NICD) is seen simultaneously with ADAM10 (Fig. 8*M*). However, this seemed not to lead to systematic induction of Notch target gene expression, which may indicate severe disturbance between activated Notch and transcriptional control of the target genes. It has been shown that HIF2α, which was stabilized in the cKO skin as a result of *Hif-p4h-2* deletion, may inhibit NICD activity by binding to its recombination signal–binding protein for immunoglobulin kappa J region–associated module domain (57, 58) and subsequently downregulate NOTCH1-dependent target genes, which is in accordance with the similar skin phenotype resulting from *Notch1* (58) and *Hif-p4h-2* deletion. Moreover, FIH has been shown to inhibit NICD, which could strengthen this hypothesis (Fig. S5, *J* and *K*). HIF1α, on the contrary, physically binds to NICD, causes its stabilization, and increases the Notch target gene expression (57, 58). From Notch receptors, only NOTCH1 is expressed abundantly in the *FoxD1*-positive HF cells (including DP cells) and thus may be able to interact directly with HIF1α and HIF2α (or FIH) in DP cells. On the other hand, many Notch pathway genes are regulated by HIF (41, 59, 60) and hence, the stabilization of HIF caused by deletion of *Hif-p4h-2* may interfere Notch signaling *via* modifying the expression of, for example, *Notch3*, *Dll1*, *Dll4*, *Jag2*, and *Hes1*. The imbalance between HIF and Notch pathways is likely to play a crucial role in cyst formation and the failure in regeneration of HFs in the cKO skin.

NOTCH1 has been implicated to be important for the differentiation of both the HF matrix cells and the cells in the IRS, especially the Henle’s layer (56), that is absent from the cKO HF cysts (Fig. 6). The IRS protects the hair and is crucial for withholding the HF structure and for the hair formation itself (61). The Henle’s cell layer of the IRS is the first HF layer to reach full keratinization and is fundamental for maintaining the HF structure (39, 40). In the cKO mice HF, Henle’s cell layer disappears when the epidermal cyst structures develop and is no longer capable of maintaining the HF integrity (Fig. 6). Our results clearly show that expression of various KRTs and KRT-related proteins, especially *Krt1*, *Krt14*, and *Krt15*, is markedly distorted in cKO skin (Fig. 5*C*). Deletion of *Krt14* is known to enhance NOTCH1 signaling, and KRT14/KRT15 heterodimers are suggested to be important for promoting and maintaining cell proliferation in the epidermal basal cell layer (33). KRT15, the marker of the HF SCs, not only is expressed in higher amounts in cKO skin but also has changed its physical localization in the cKO HF (Fig. 5*B*). This is interesting since Notch signaling is also considered to be involved in determining the differentiation programs of the HF SCs (51, 62). Major regulators of the HF cycling are KRT15^+^ SCs that are located at the lowest part of the permanent HF in the bulge region, and which can give rise to a new HF after signaling from the DP cells (37). In the cKO HF, the intensity of KRT15 staining was much lower and restricted only to a small area above the DP, whereas in controls, KRT15^+^ cells traveled toward the skin surface along the edges of the HF (Fig. 5*B*), which suggests that the SC differentiation, proliferation, and/or migration may be interrupted in cKO mice.

While Notch signaling regulates skin cell fate decision in the HF bulge SCs (63), TGFβ signaling is involved in both morphogenesis and HF cycling. TGFβ1 is especially important in the anagen–catagen transition as well as in the telogen–anagen transition (13, 47, 64). Hypoxia has been shown to regulate matrix proteinases and thrombospondin, whose activation may lead to the activation of TGFβ, and this phenomenon has been suggested to be HIF mediated in hepatocytes (65). Furthermore, HIF1α accumulation in alveolar macrophages is associated with TGFβ1-induced plasminogen activator inhibitor 1 production and is reversibly inhibited by HIF1α silencing (66). Hypoxia also affects directly the SMAD2–SMAD3 complex by enhancing its transfer to the nucleus in human dermal fibroblasts and subsequently drives them into transition to myofibroblasts (67). TGFβ ligands are expressed in the DP cells, which in turn activate the overlying basal epithelial cell TGFβ signaling during catagen (68). Increased TGFβ1 signaling has been shown to induce apoptosis in the HF (48). We observed significantly upregulated canonical TGFβ signaling in the cKO keratinocytes relative to the control (Fig. 7, *F* and *G*), which was in accordance with upregulation of some of the TGFβ target genes in the mutant skin (Fig. 7, *B* and *C*). Although no statistically significant increase in the apoptosis of skin cells was observed in the mutant mice (Fig. S4, *C* and *D*), differences in the mRNA level of the apoptosis-related *Bnip3* were detected between the genotypes suggesting potential disturbances in the regulation of apoptosis (Fig. S4*E*). Furthermore, the basal epithelial cells and macrophages have been shown to act as phagocytes and clear away apoptotic cells in the HFs (68), and induction of *Bnip3* has been associated to autophagy of the keratinocytes themselves (69), which may hamper detection of changes in apoptosis. Interestingly, it has also been shown that the TGFβ-induced regression phase reduces the SC pool (68). TGFβ1 is also an important factor in the regulation of the HF bulge SC differentiation (61, 62) and has been shown to control mesenchymal SC differentiation to smooth muscle cells *via* regulation of the Jag1–Notch signaling in vascular development (70). Furthermore, in line with our current findings, a recent study showed that endothelial cell–specific *Hif-p4h-2* deletion in the lungs increases both TGFβ and NOTCH3 signaling because of HIF2α stabilization (71).

**Figure 9:**
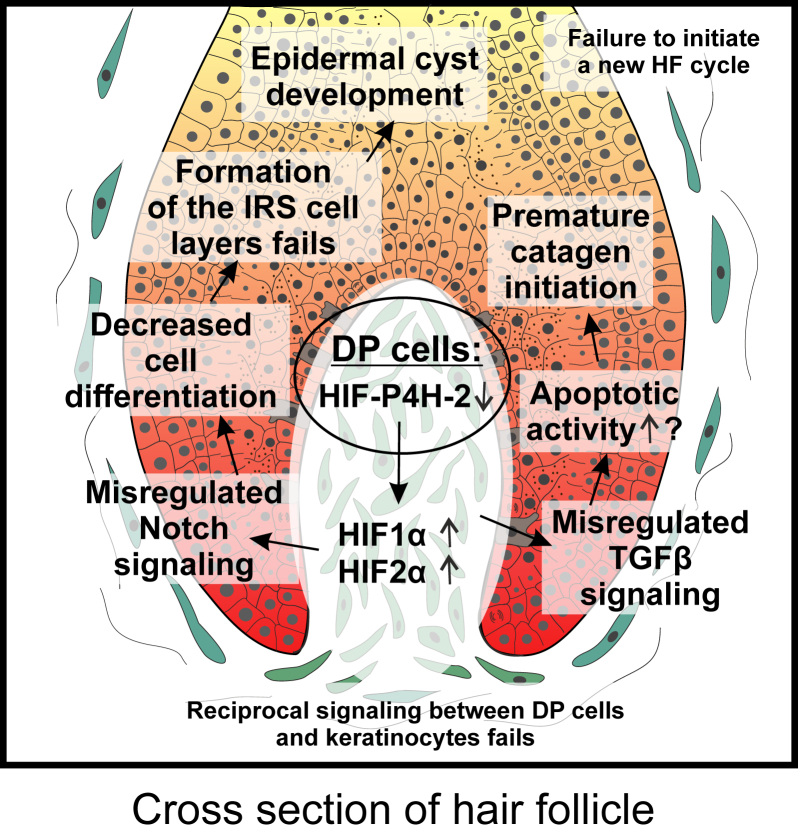
**Schematic summary of the effects of HIF-P4H-2 depletion on HF development.** As HIF-P4H-2 is lacking in the DP cells, HIF1 and HIF2 are stabilized and induce expression of HIF target genes. This also affects Notch and TGFβ signaling leading to disturbed homeostasis between the three signaling pathways. As a result, the receptor–ligand interactions between the DP cells and keratinocytes are altered, the cell differentiation fails, the formation of the supporting IRS layers fails, and an epidermal cyst develops. Furthermore, apoptotic activity may be increased in the keratinocytes. Altogether, this leads to premature catagen initiation. DP, dermal papilla; HF, hair follicle; HIF, hypoxia-inducible factor; HIF-P4H, HIF prolyl 4-hydroxylase; IRS, inner root sheath; TGFβ, transforming growth factor β.

In conclusion, our results show that *Hif-p4h-2* expression in the mesenchymal *FoxD1*-lineage cells has a fundamental physiological role in normal development of the truncal hair and HFs, and the lack of *Hif-p4h-2* in the *FoxD1*^*+*^ dermal fibroblasts and DP cells causes congenital alopecia. The most superficial parts of the skin have the most severe physiological hypoxia, as they are furthest away from the blood vessels that are located in the skin dermis, and HIF has been reported to be endogenously stabilized in these parts of the skin (14, 15, 16). As the HF morphogenesis proceeds, the dermal placode cells, that later develop into DP cells, travel closer to the hypodermal area, where there is a higher oxygen concentration, and thus, the endogenous HIF stabilization should normally be reduced or disappear in the DP cells. The highest oxygen concentration and hence lowest HIF amount in the DP cells should be when the HF is at its longest in the end of HF morphogenesis, before the beginning of the first HF cycle, or catagen. However, in our cKO mice, HIF is constantly stabilized in the DP cells regardless of the oxygen concentration, which results in activation of the hypoxia response pathway and misregulation of Notch and TGFβ signaling (Fig. 9). This leads to an imbalance in the reciprocal signaling between the DP cells and the bulge SCs causing reduced differentiation of the HF keratinocytes. Because of the lack of differentiation, HFs cannot maintain their proper structure, which results in HF cyst formation and poor hair production. In the poorly differentiated epidermal and HF cells, KRT expression is significantly increased and the hair shaft production is disturbed resulting in alopecia in the mutant mice.

Several substrates besides HIF have been proposed for the HIF-P4Hs (9, 72). However, our recent study using rigorous biochemical analyses did not provide evidence for a wide range of non-HIF substrates under conditions that resulted in highly effective hydroxylation of HIF peptides (9), but we cannot exclude the possibility that hydroxylation of non-HIF substrates might require additional, yet unknown factors, and that such a hydroxylation could take place in cells. Therefore, we consider it possible but unlikely that certain Notch or TGFβ pathway protein could be direct hydroxylation targets of HIF-P4H-2. Instead, several publications have reported on reciprocal effects between the HIF, Notch, and TGFβ signaling pathways, and our current conclusion thus is that the observed upregulation of HIF1 and HIF2 in the current model leads to an imbalance between HIF, Notch, and TGFβ signaling homeostasis required for normal HF development.

## Experimental procedures

### Mice

In brief, a conditional *Hif-p4h-2* targeting construct resulting in the deletion of exon 3 encoding two catalytically critical residues, a histidine and an arginine required for binding of Fe^2+^ and 2-oxoglutarate (2), respectively, was generated from a 7 kb genomic clone from Lambda FIX Library (Stratagene) (Fig. S1*A*). The targeting construct was electroporated into mouse embryonic stem (ES) cells, and positively targeted ES cell clones were identified by Southern blotting (Fig. S1*B*). Correctly targeted ES clones were used to generate chimeric mice *via* blastocyst injections in the BioCenter Oulu Transgenic Core Facility. The chimeras were crossed with C57/Bl6N mice, and the offspring were genotyped by PCR (Fig. S1*C*). To inactivate *Hif-p4h-2* in *FoxD1*-lineage cells, the *Hif-p4h-2*^*loxP/loxP*^ mice were bred with a *FoxD1-Cre* mouse line (Jackson B6; 129S4-Foxd1^tm1(GFP/cre)Amc^/J) (73) purchased from The Jackson Laboratory. Deletion of exon 3 and reduced amount of HIF-P4H-2 were confirmed by PCR and Western blotting (Fig. S1, *D* and *E*). Both female and male mice were used in the study since they expressed the same phenotype. The double *Cre*-reporter Rosa26^*mT/mG*^ mice (The Jackson Laboratory; catalog no.: 007676) (74) were a kind gift from Prof Seppo Vainio, University of Oulu. The Animal Experiment Board of Finland, following the regulations of the European Union Directive 86/609/EEC, the European Convention ETS123, and the national legislation of Finland, approved the animal experiments in this study. Recommendations concerning laboratory animal experiments and handling given by the Federation of European Laboratory Animal Science Associations and the Finnish and European Union legislations were followed.

### Tissue preparation and processing

Mouse skin biopsies were taken as indicated (Fig. 1*A*). Samples were fixed overnight in 10% phosphate-buffered formalin and embedded in paraffin. Five micrometer sections were cut with Microm HM355S Microtome (Thermo Fisher Scientific). For cryosections, cranial skin biopsies were prepared from the cranial area (frontal to occipital area) at P21, and dorsal skin biopsies were prepared from P14, P21, and P27 mice. In brief, the tissue was coated with Tissue-Tek optimal cutting temperature embedding compound (Sakura; catalog no.: SA62550), immediately immersed in liquid nitrogen, and placed in a Tissue-Tek Cryomold (Sakura; catalog no.: SA62534). Cryoblocks were cut with Cryotome Leica CM3050S.

### Immunohistochemistry, immunofluorescence, and morphometric analysis

Paraffin sections were stained with hematoxylin and eosin. For immunohistochemical and immunofluorescent stainings, the samples were pretreated with citric acid before staining with antibodies shown in Table S2. Apoptosis was analyzed using TUNEL assay by an *In Situ* Cell Death Detection kit (Roche; catalog no.: 11684795910). As counterstain for immunohistochemical and immunofluorescent stainings, Hoechst or 4′,6-diamidino-2-phenylindole staining was used. For the analysis of *Rosa26*^*mT/mG*^ expression, *Rosa26*^*mTmG*^*;FoxD1*^*cre/+*^ dorsal skin cryosections were air-dried for 1 h, fixed with 10% phosphate-buffered formalin for 10 min, washed with isopropanol and counterstained with Hoechst diluted in isopropanol, and mounted. Morphometric analyses were performed with Adobe Photoshop CS software from 4 to 18 sections/mouse and from 4 to 35 mice per genotype as indicated in the figure legends. Time points included in the analyses ranged from E18.5 to P29. In proliferating cell nuclear antigen and TUNEL morphometric analyses of the HS, KRT mass inside the cysts and sebaceous glands were not calculated to the HF area. The number of pSMAD2^+^ HF keratinocytes per total 4′,6-diamidino-2-phenylindole^+^ cell number was calculated. The visual field analyzed was a 10× magnification field.

### Western blotting

Protein lysates were prepared from snap-frozen dorsal skin with urea buffer (8 M urea, 40 mM Tris-HCl, 2.5 mM EDTA, and pH 8.0) containing phosphatase (PhosSTOP; Roche) and protease inhibitors (cOmplete Protease Inhibitor Cocktail Tablet EDTA-free; Roche). The lysates were analyzed by Western blotting with antibodies shown in Table S2.

### Quantitative RT–PCR

Total RNA was isolated from dorsal skin using TriPure Isolation Reagent (Roche; catalog no.: 11667157001) and treated with DNase I (Thermo Fisher Scientific; catalog no.: EN0521). Reverse transcription of 1 μg RNA/20 μl was performed with the iScript complementary DNA Synthesis Kit (Bio-Rad; catalog no.: 1708890). Quantitative PCR was performed with iTaq Universal SYBR Green Supermix (Bio-Rad; catalog no.: 1725120). To minimize the variation of the housekeeping genes, we used the geometrical mean of β-actin and GAPDH for normalization of the data (75). The data values are shown as relative levels normalized to the E18.5 control expression levels. Quantitative PCR primers are shown in Table S3. The following number of samples was analyzed at different time points: E18.5 control/cKO (n = 6/6), P4 (n = 5/5), P8 (n = 4/4), P14 (n = 6/6), P16 (n = 5/5), P18 (n = 5/4), P21 (n = 7/5), and P24 (n = 4/7).

### Electron microscopy

TEM and scanning electron microscopy were performed in the BioCenter Oulu Electron Microscopy Core Facility. For TEM analysis, P14 and P24 dorsal skin biopsies were processed and analyzed as described (76).

For scanning electron microscopy analysis, P24 dorsal skin biopsies were fixed in 2.5% glutaraldehyde in 0.1 M phosphate buffer, dehydrated in graded ethanol series, and dried using critical point dryer (K850; Quorum Technologies). Dried samples were attached to aluminum specimen mount using double-sided carbon tape and coated with 5 nm of platinum (Q150T ES; Quorum Technologies). Samples were examined in Σigma HD VP scanning electron microscopy (Carl Zeiss Microscopy).

### Statistical analyses

The statistical analyses were performed using Student’s *t* test or Welch’s *t* test. The data are shown as the means ± SD. Values of *p* < 0.05 were considered statistically significant (∗*p* < 0.05, ∗∗*p* < 0.01, and ∗∗∗*p* < 0.001).

## Data availability

All data are available in the main article or the supporting information.

## Supporting information

This article contains supporting information (1, 2, 3, 4, 5, 6, 7, 26, 27).

## Conflict of interest

J. M. reports that financial support was provided by 10.13039/100006591FibroGen, Inc and also reports a relationship with FibroGen, Inc that includes equity or stocks. All other authors declare that they have no conflicts of interest with the contents of this article.
