# Supplementary material for: Deletion of hypoxia-inducible factor prolyl 4-hydroxylase 2 in FoxD1-lineage mesenchymal cells leads to congenital truncal alopecia
Source: J Biol Chem. 2022 Mar 2;298(4):101787. doi: 10.1016/j.jbc.2022.101787 (PMC8988008; doi:10.1016/j.jbc.2022.101787)
Supplement: Supplemental Figures S1–S5 and Table S1–S3 [file mmc1.pdf]

Supporting information

Deletion of hypoxia-inducible factor prolyl 4-hydroxylase 2 in *FoxD1*-lineage mesenchymal cells leads to congenital truncal alopecia

Ann-Helen Rosendahl[1,2,3], Mia Monnius[1,2,3], Anu Laitala[1,2,3], Antti Railo[1,2,3], Ilkka Miinalainen[4], Ritva Heljasvaara[1,2,3], Joni M. Mäki[1,2,3*] and Johanna Myllyharju[1,2,3*]

[1]Oulu Center for Cell-Matrix Research, University of Oulu, Oulu, FIN-90014, Finland
[2]Biocenter Oulu, University of Oulu, Oulu, FIN-90014, Finland
[3]Faculty of Biochemistry and Molecular Medicine, University of Oulu, Oulu, FIN-90014, Finland
[4]Biocenter Oulu, Electron Microscope Core Facility, University of Oulu, Oulu, FIN-90014, Finland

*Shared last authors.

Corresponding authors: joni.maki@oulu.fi , johanna.myllyharju@oulu.fi

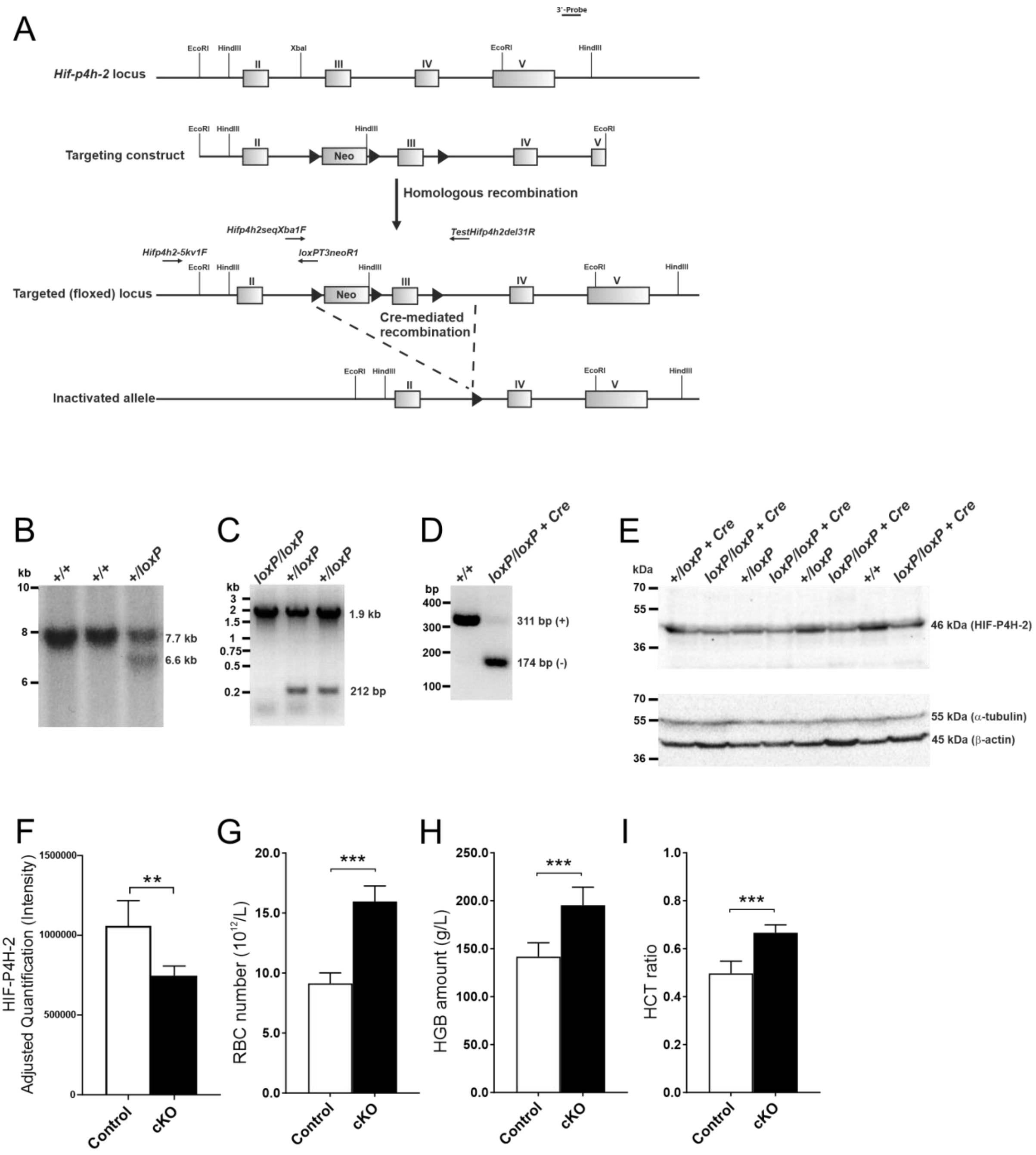

Figure S1. Generation of *Hif-p4h-2*$^{loxP/loxP}$*;FoxD1*$^{Cre/+}$ (cKO) knock-out mice. (A) Schematic representation of the gene targeting strategy. The organization of the wild-type (wt) *Hif-p4h-2* gene, the targeting construct, and the structure of the locus following gene targeting are shown. Exons are depicted as gray boxes and numbered. *LoxP*-sequences are depicted as black arrowheads. The locations of PCR primers, Hind-III recognition sites and the 3'probe used for genotyping are shown in the picture. (B) Targeting was identified in ES cell lines by using the 3' probe and Hind-III digestion in Southern hybridization, where 7.7-kb wt (+) and 6.6-kb *Hif-p4h-2* conditional alleles (*loxP*) were identified, as expected. (C) Genotyping by PCR using the primer pairs Hifp4h2seqXba1F/TestHifp4h2del31R and Hifp4h2-5kv1F/loxPT3neoR1 from the F2 generation showed 212-bp and 1.9-kb bands for wt (+) and *Hif-p4h-2* conditional (*loxP/loxP*) alleles, respectively. (D) *FoxD1Cre*-mediated deletion of the

Hif-p4h-2 exon 3 was confirmed at the mRNA level from kidney by PCR primers mHifp4h2ex2F and mHifp4h2ex4R from exons 2 and 4, respectively, resulting in 311-bp wt (+) and 174-bp mutant (-) bands, as expected. (E) Western blotting and (F) quantification of HIF-P4H-2 from control and *Hif-p4h-2*$^{loxP/loxP}$*;FoxD1*$^{Cre/+}$ skin. Note that the same Western blot was used for analysis of HIF-P4H-2, FIH (Fig. S5K) and the loading controls with stripping in between the primary antibody treatments. Therefore, the β-actin/α-tubulin loading control image is reused in Fig. S5K. (G-I) Blood analysis of the control and cKO mice showed increased levels of red blood cells (RBC), hemoglobin (HGB) and hematocrit (HCT). N = 6-7 per genotype Data are presented as mean ± S.D. ** P<0.01; *** P<0.001

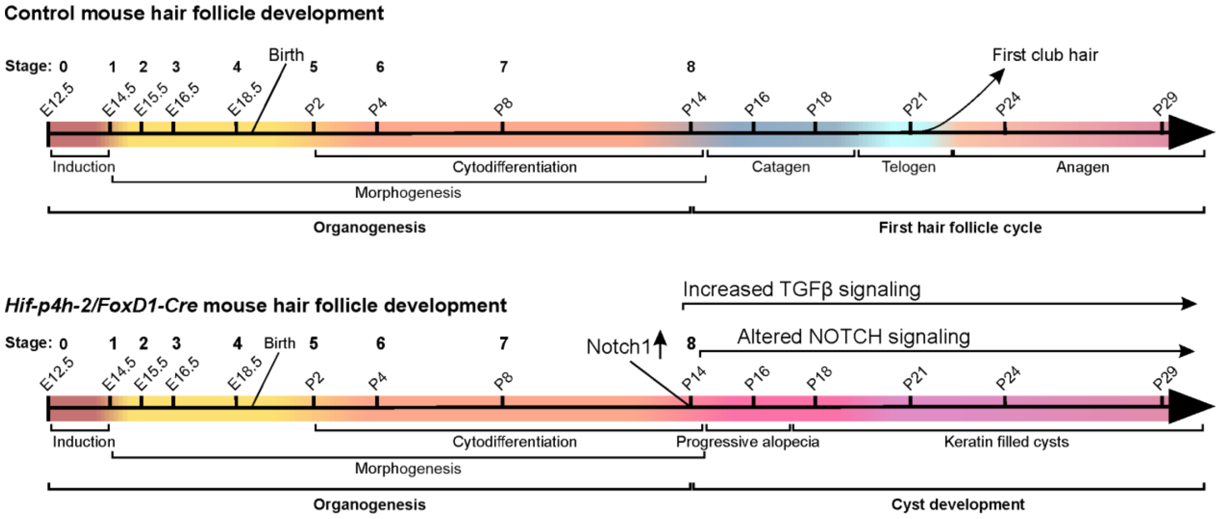

Figure S2. Schematic representation of the timeline of HF development in control and cKO mice. A progressive alopecia starts at P15 in the cKO mice and evolves to keratin-filled epidermal cysts. TGFβ signaling is upregulated and Notch signaling is altered starting from P14 in the cKO mice relative to the control.

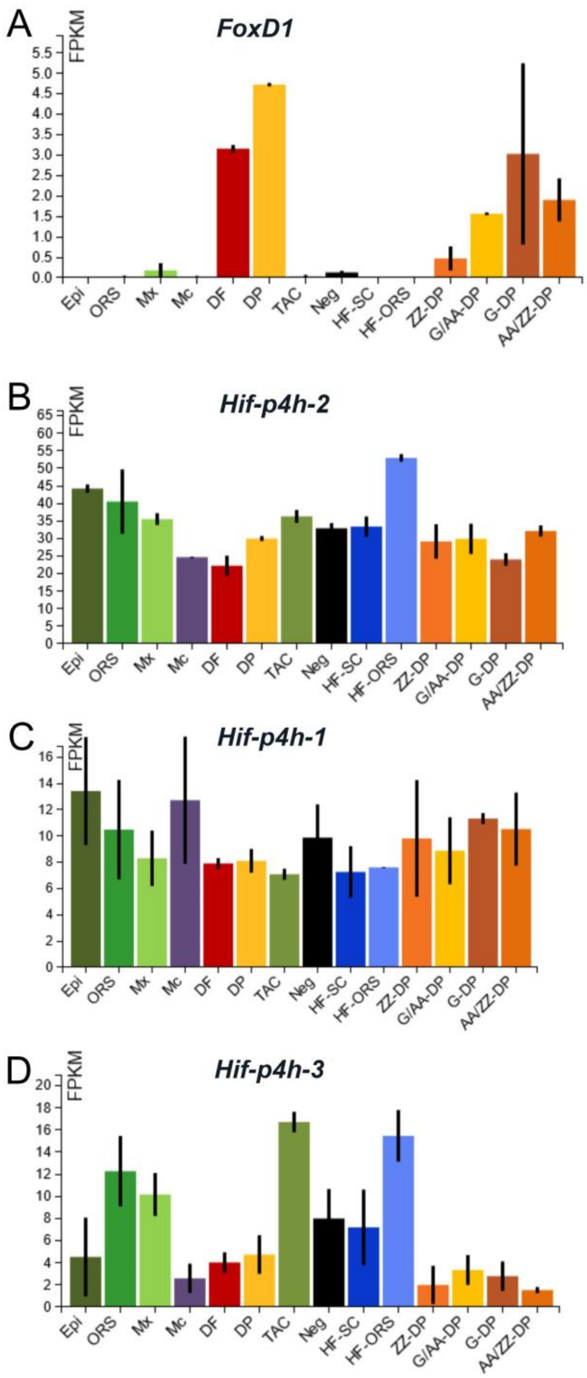

Figure S3. The expression of (A) *FoxD1* and HIF-P4H isoenzymes (B) *Hif-p4h-2*, (C) *Hif-p4h-1* and (D) *Hif-p4h-3* in mouse (P5) hair follicle and skin cell types. FPKM, Fragments Per Kilobase of transcript per Million mapped reads; Epi, epidermis; ORS, outer root sheat; Mx, matrix; Mc, melanocytes; DF, dermal fibroblasts; DP, dermal papilla; TAC, transit amplifying cells; Neg, population containing all remaining skin cells; HF-SC, bulge stem cell precursors; HF-ORS, remainder of the ORS; ZZ-DP, zigzag DP; G/AA-DP, a complementary mixture of guard/awl/auchene hair; G-DP, guard DP; AA/ZZ-DP, a mixture of AA-DPs and ZZ-DPs. See further details from Hair-gel.net and corresponding references (26,27).

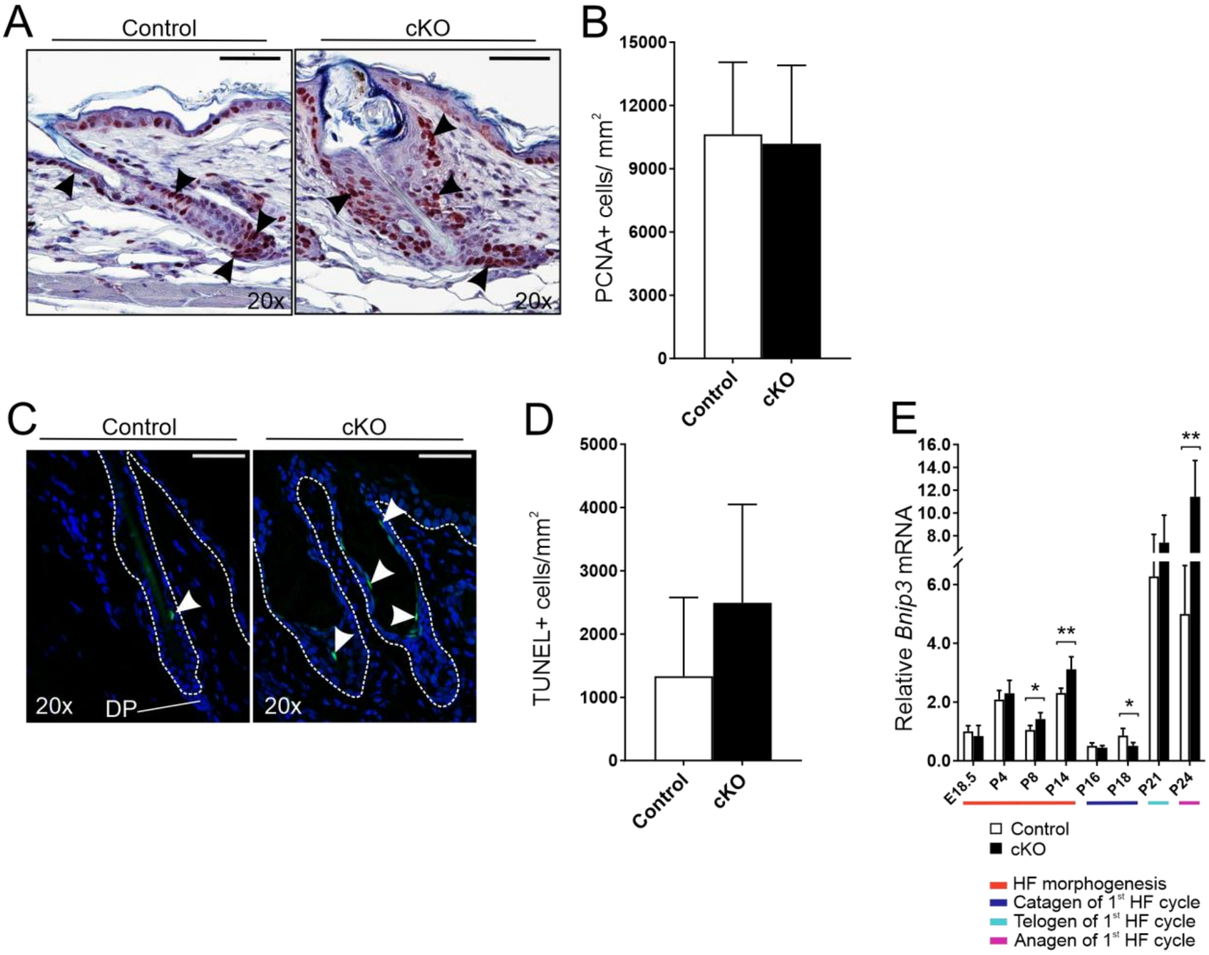

Figure S4. Apoptosis and proliferation of cells is unaffected in the cKO mouse skin. (A) Analysis of proliferating cells (indicated by arrowheads) in the skin (P21) by immunohistochemical staining of PCNA. Scale bars: 50 µm. (B) Morphometric analysis of $PCNA^+$ cells in the skin (P21). Control (n = 5), cKO (n = 5). Scale bars: 50 µm. (C) Analysis of apoptotic cells (indicated by arrowheads) in the skin (P21) by TUNEL staining. (D) Morphometric analysis of apoptotic cells in the skin (P21). Control (n = 5), cKO (n = 5). (E) qPCR analysis of *Bnip3* mRNA expression at indicated time points. The colours beneath the bar charts indicate the HF cycle stages, n = 4-7 per genotype. Data are presented as mean ± S.D. * P<0.05; ** P<0.01.

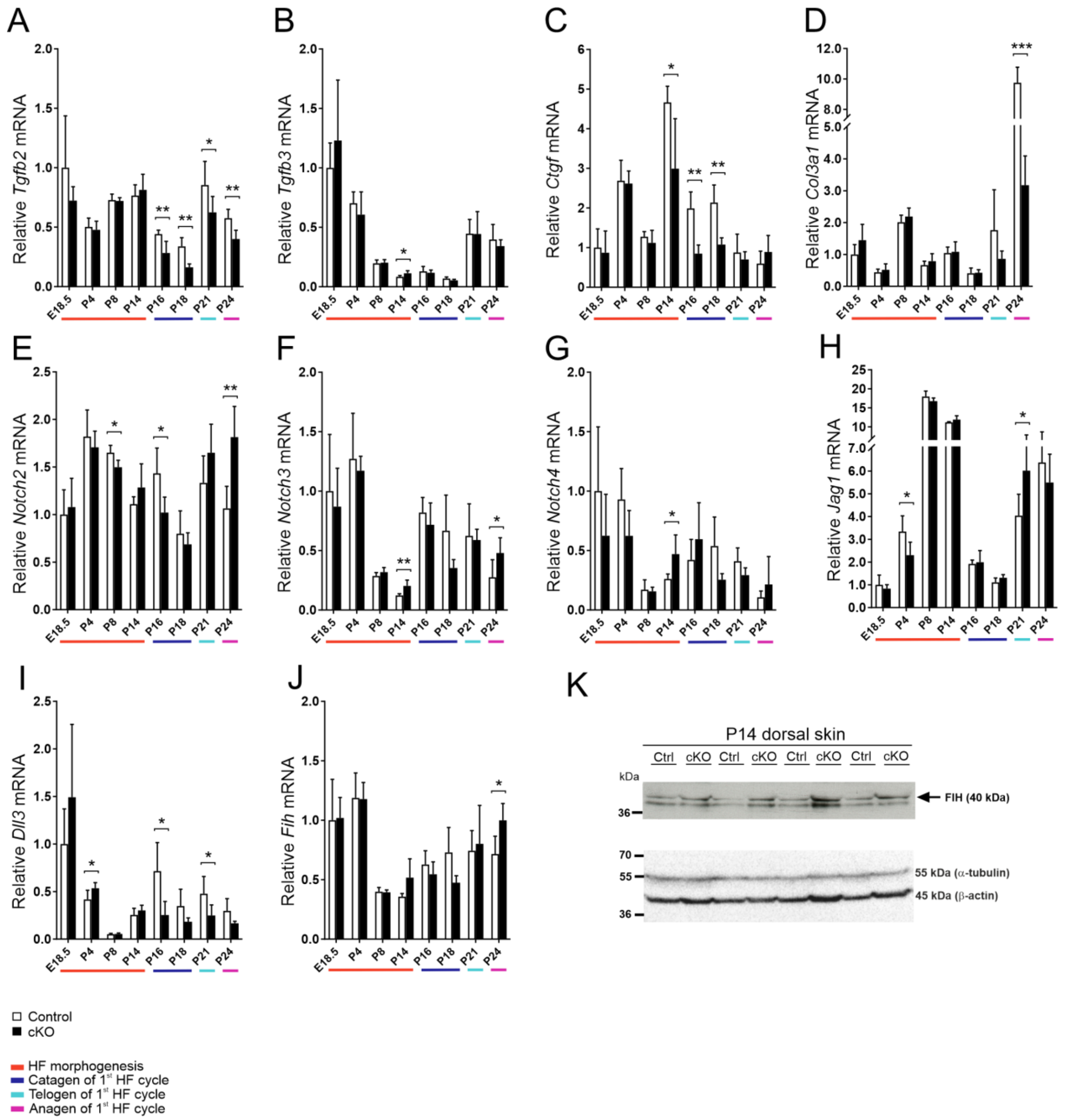

Figure S5. Expression of TGFβ and Notch pathway related genes is altered in the cKO mice. qPCR analysis of the mRNA expression of the TGFβ isoforms *Tgfb2* (A) and *Tgfb3* (B); TGFβ target genes *Ctgf* (C) and *Col3a1* (D)*;* NOTCH isoforms *Notch2* (E), *Notch3* (F) and *Notch4* (G); and NOTCH ligands *Jag1* (H) and *Dll3* (I); and *Fih* (J) at the indicated time points in the skin. The colours beneath the bar charts indicate the HF cycle stages, n = 4-7 per genotype. (K) Western blot analysis of FIH in P14 dorsal skin samples. Note that the same Western blot was used for analysis of HIF-P4H-2 (Fig. S1E), FIH and the loading controls with stripping in between the primary antibody treatments. Therefore, the β-actin/α-tubulin loading control image from Fig. S1E is reused in Fig. S5K. The expected approximate molecular weights of FIH, β-actin and α-tubulin are 40, 45 and 55 kDa, respectively. Data are presented as mean ± S.D. * P<0.05; ** P<0.01; *** P<0.001.

Table S1. Nomenclature of HIF prolyl 4-hydroxylases (see references 1-7 for further details). The isoenzyme studied in this study is denoted in bold.

HIF-P4H-1	PHD1	EGLN2	HPH3
**HIF-P4H-2**	**PHD2**	**EGLN1**	**HPH2**
HIF-P4H-3	PHD3	EGLN3	HPH1

Table S2. Antibodies and other reagents used for immunostaining or Western blot analyses.

Name	Code	Company	Dilution
Adam10	ab1997	Abcam	WB 1:1000
Activated Notch1	ab8925	Abcam	WB 1:500
Cy3	715-165-150<br>711-165-152<br>712-165-150	Jackson Immunoresearch	IF 1:300
Keratin 1	ab24643	Abcam	IF 1:100
Keratin 15	ab2414	Abcam	IF 1:100
DAPI	D9542	Sigma	IF 1:500
FIH-1/HIF-1AN	NB100-428SS	Novus Biologicals	WB 1:500
HDAC1	#5356	Cell Signaling Technology	WB 1:1000
Hes1	ab71559	Abcam	WB 1:1000
HIF1 alpha	NB100-479	Novus Biologicals	WB 1:500
HIF2 alpha	GTX30114	GeneTex	WB 1:500
Hoechst	33258	Sigma Aldrich	IF 1:10000
Keratin 5	PRB-160P	Biolegend	IF 1:100
Loricrin	PRB-145P	Biolegend	IF 1:200
Mac-3	550292	BD Pharmingen™	IHC 1:100
Notch1	ab27526	Abcam	WB 1:500
PCNA	sc-56	Santa Cruz Biotechnology	IHC 1:100
EGLN1/PHD2	NB100-2219	Novus Biologicals	WB 1:500
Phospho-Smad2 (Ser465/467)	#3101	Cell Signaling Technology	IF 1:100
α-Tubulin	B-6199	Sigma Aldrich	WB 1:20000

Table S3. PCR primers

Name	Gene	Oligo direction	Oligo sequence
Adam8	ADAM metallopeptidase domain 8	Forward	CCCAAGACCCATAGTGAAACCAA
		Reverse	CTTTGGGGCATAAACAGGAACTG
Adam10	ADAM metallopeptidase domain 10	Forward	AACATCAGCTTCATGGTGAAACG
		Reverse	CCAGGAACTTCTCCACACCAATA
Adam17	ADAM metallopeptidase domain 17	Forward	GGTTCTAGCCCACATAGGAGATG
		Reverse	CCTCCAAAGTGGCTCTACGTTAT
β-Actin	β-Actin	Forward	AGAGGGAAATCGTGCGTGAC
		Reverse	CAATAGTGATGACCTGGCCGT
Bnip3	BCL2/adenovirus E1B interacting protein 3	Forward	GCTCCAAGAGTTCTCACTGTGAC
		Reverse	GTTTTTCTCGCCAAAGCTGTGGC
Col3a1	Collagen type III alpha 1 chain	Forward	CTGTAACATGGAAACTGGGGAAA
		Reverse	CCATAGCTGAACTGAAAACCACC
Cre	Cre recombinase (used for genotyping)	Forward	GCACGTTCACCGCATCAAC
		Reverse	CGATGCAACGAGTGATGAGGTTC
Ctgf	Connective tissue growth factor	Forward	GGGCCTCTTCTGCGATTTC
		Reverse	ATCCAGGCAAGTGCATTGGTA
Dll1	Delta like canonical Notch ligand 1	Forward	CAGGACCTTCTTTCGCGTATG
		Reverse	AAGGGGAATCGGATGGGGTT
Dll3	Delta like canonical Notch ligand 3	Forward	GTCATACCAGCCCCTTCCATTTA
		Reverse	GCGATGATAGAGAAGGGACAAGA
Dll4	Delta like canonical Notch ligand 4	Forward	TCCCAGGGACTCTATGTACCAAT
		Reverse	CTGAGTAGGCTCCTGCCTTATAC
Eln	Elastin	Forward	TGTCCCACTGGGTTATCCCAT
		Reverse	CAGCTACTCCATAGGGCAATTTC
Eno1	Enolase 1	Forward, reverse	QuantiTect Mm_Eno1_1_SG (QT00260442)
Fgf7 (Kgf)	Keratinocyte growth factor	Forward	AGCTGTTCCAAACAGAACAAAAGT
		Reverse	ACCAATAACACGATTCCTCCTTCA
Fih	Factor inhibiting Hif	Forward	ATGAGTCCCAGCTACGAAGTTAC
		Reverse	CAGTGCAGGATACACAAGGTTTG
Flg	Filaggrin	Forward	AGATGTCCGCTCTCCTGGAA
		Reverse	TGGATTCTTCAAGACTGCCTGTA
Fn1	Fibronectin	Forward, reverse	QuantiTect Mm_Fn1_1_SG (QT00135758)
GAPDH	Glyceraldehyde-3-phosphate dehydrogenase	Forward	TGTGTCCGTCGTGGATCTGA
		Reverse	TTGCTGTTGAAGTCGCAGGAG
Hes1	Hes family bHLH transcription factor 1	Forward	CGGCATTCCAAGCTAGAGAAGG
		Reverse	GGTAGGTCATGGCGTTGATCTG
Hes5	Hes family bHLH transcription factor 5	Forward	CATCAACAGCAGCATAGAGCAG
		Reverse	GCGAAGGCTTTGCTGTGTTTCA
Hey1	Hairy/enhancer-of-split related with YRPW motif 1	Forward	TGAGCTGAGAAGGCTGGTAC
		Reverse	ACCCCAAACTCCGATAGTCC

Table S3. PCR primers continued

Name	Gene	Oligo direction	Oligo sequence
Hey2	Hairy/enhancer-of-split related with YRPW motif 2	Forward	GACTTCATGAGCATTGGATTCCG
		Reverse	CAGGTGCTGAGATGAGAGACAAG
HeyL	Hairy/enhancer-of-split related with YRPW motif-like	Forward	TGGGTCAAGAGAACGATCTTAGC
		Reverse	CTATGATCCCTCTGCGCTTCTTC
Hif-p4h-1	HIF prolyl 4-hydroxylase 1	Forward	AGAACTGGGATGTTAAGGTGCAT
		Reverse	GAAAATGAGCAACCGGTCAAAGAG
Hif-p4h-2	HIF prolyl 4-hydroxylase 2	Forward	GAGATGGAAGATGCGTGACA
		Reverse	TTGCCTTCTGGAAAAATTCG
Hifp4h2-5KV1F	HIF prolyl 4-hydroxylase 2 (used for genotyping)	Forward	ACCAACCATTCCTAGAGAGTCATC
Hifp4h2seqXba1F	HIF prolyl 4-hydroxylase 2 (used for genotyping)	Forward	GCTCAGGAGCAGCAGTGTG
Hif-p4h-3	HIF prolyl 4-hydroxylase 3	Forward	CTTATTCAGGTAGTAGATACAGGTGATACA
		Reverse	GCTGGGCAAATACTATGTCAAG
Hk2	Hexokinase 2	Forward, reverse	QuantiTect Mm_Hk2_1_SG (QT00155582)
Jag1	Jagged 1	Forward	CCTCGGGTCAGTTTGAGCTG
		Reverse	CCTTGAGGCACACTTTGAAGTA
Jag2	Jagged 2	Forward	TGGCTGTCACCGAGGTCAA
		Reverse	ACGTTCTTTCCTGCGCTTTC
Krt1	Cytokeratin 1	Forward	AGCTGAATCGAATGATCCAGAGA
		Reverse	TGCTGTATTTGGGAGATCTGCTT
Krt5	Cytokeratin 5	Forward	GGTGGAGGACTACAAGAACAAGT
		Reverse	GCATCCACATCCTTCTTCAACAT
Krt10	Cytokeratin 10	Forward	AGGACCAAGATACTAACAAAACCAA
		Reverse	AGTGGCCCGTATGAAGAGACT
Krt14	Cytokeratin 14	Forward	CAAAGACTACAGCCCCTACTTCA
		Reverse	CTCAAACTTGGTCCGGAAGTCAT
Krt15	Cytokeratin 15	Forward	GAGGTGAAGATCCGAGATTGGTA
		Reverse	CCAGAATTTTGTCCCGGATCTCT
Ldha	Lactate dehydrogenase A	Forward, reverse	QuantiTect Mm_Ldha_1_SG (QT01045492)
Lor	Loricrin	Forward	CATGAATTTGCCTGAGGTTTCCA
		Reverse	GGGAGGTAGTCATTCAGAAACCA
loxPT3neoR1	Genotyping primer	Reverse	GCTATACGAAGTTATTAGGTCC
mHifp4h2ex2F	Genotyping primer	Forward	GCCATGGTTGCTTGTTACCC
mHifp4h2ex4R	Genotyping primer	Reverse	ACTTTAGCTCTCGCTCGCTC
Ngf	Nerve growth factor	Forward	GGGAGCGCATCGAGTTTTG
		Reverse	CCAGTATAGAAAGCTGCGTCCTT
Notch1	Notch 1	Forward	GATGGCCTCAATGGGTACAAG
		Reverse	TCGTTGTTGTTGATGTCACAGT
Notch2	Notch 2	Forward	GAGAAAAACCGCTGTCAGAATGG
		Reverse	GGTGGAGTATTGGCAGTCCTC

Table S3. PCR primers continued

Name	Gene	Oligo direction	Oligo sequence
Notch3	Notch 3	Forward	AGATGCACTGGGAATGAAGAACA
		Reverse	GCTCCTCTACCTTCAGTCTCTTG
Notch4	Notch 4	Forward	GAACGCGACATCAACGAGTG
		Reverse	GGAACCCAAGGTGTTATGGCA
Pdk1	Puryvate dehydrogenase kinase 1	Forward, reverse	QuantiTect Mm_Pdk1_1_SG (QT00116396)
Postn	Periostin	Forward, reverse	QuantiTect Mm_Postn_1_SG (QT00150759)
Serpine1 (Pai-1)	Plasminogen activator inhibitor-1	Forward, reverse	QuantiTect Mm_Serpine1_1_SG (QT00154756)
Slc2a1	Glucose transporter 1	Forward, reverse	QuantiTect Mm_Slc2a1_1_SG (QT01044953)
TestHifp4h2del31R	HIF prolyl 4-hydroxylase 2 (used for genotyping)	Reverse	CTGGCCCTTGTGTTTCCGAC
Tgfb1	Transforming growth factor β1	Forward	GAGCCCGAAGCGGACTACTA
		Reverse	TGGTTTTCTCATAGATGGCGTTG
Tgfb2	Transforming growth factor β2	Forward	AGTTTACACTGCCCCTGCTG
		Reverse	AGAGGTGCCATCAATACCTGC
Tgfb3	Transforming growth factor β3	Forward	CACCACAACCCACACCTGAT
		Reverse	CAGGTTGCGGAAGCAGTAAT
Tnfa	Tumor necrosis factor α	Forward	GGTGCCTATGTCTCAGCCTCTT
		Reverse	GCCATAGAACTGATGAGAGGGAG
Vegfa	Vascular endothelial growth factor A	Forward	CCACGTCAGAGAGCAACATCA
		Reverse	TCATTCTCTCTATGTGCTGGCTTT
